# Association of physical fitness with cognitive function in the community-dwelling older adults

**DOI:** 10.1186/s12877-022-03564-9

**Published:** 2022-11-16

**Authors:** Xiaoguang Zhao, Huiming Huang, Chenya Du

**Affiliations:** 1 Faculty of Sport Science, Ningbo University, No. 818 Fenghua Road, Jiangbei District, Ningbo, 315211 Zhejiang China; 2 Research Academy of Grand Health, Ningbo University, No. 818 Fenghua Road, Jiangbei District, Ningbo, 315211 Zhejiang China

**Keywords:** Cognitive function, Physical activity, Physical fitness, The community-dwelling older adults

## Abstract

**Background:**

Cognitive function generally declines with the aging process. Although the association of physical fitness with cognitive function has been proved, how many and how well the physical fitness components are linked to cognitive function is not clear. This study aimed to examine the association of physical fitness with cognitive function, and find out which aspects of physical fitness components are the most closely related to cognitive function in community-dwelling older adults.

**Methods:**

This cross-sectional study was conducted from March to July 2019. The sample consisted of 107 older people in the community with a mean age of 71.7 ± 5.0 years. The cognitive function of the participants was measured by a Chinese version of the Mini-Mental State Examination (MMSE). Several physical fitness items including grip strength, 5-repetition sit-to-stand, timed up and go, sit and reach, one-leg balance with the eye open, and 6-min walk were measured to reflect muscle strength, muscle endurance, agility, flexibility, balance, and cardiopulmonary endurance, respectively.

**Results:**

The correlation analysis showed that the grip strength and the 6-min walk were positively related to cognitive function (*r* = 0.42 and 0.35, *P* < 0.05), while the 5-repetition sit-to-stand was negatively associated with cognitive function (*r* = -0.43, *P* < 0.01) adjusting for sex, age and years of education. It was also found that the mean values of physical fitness items including grip strength and 6-min walk were significantly lower, and 5-repetition sit-to-stand and timed up and go were significantly greater in the older adults with cognitive impairment (MMSE score < 27) than those in the normal older adults (MMSE score ≥ 27) (*P* < 0.05). Stepwise regression analysis revealed that age, together with physical fitness items including grip strength and 6-min walk can explain the cognitive function in older adults.

**Conclusion:**

The findings suggest that there is an association between physical fitness and cognitive function, and the grip strength and 6-min walk can help explain the cognitive function in community-dwelling older adults. More attention needs to be paid to the increase in physical fitness for preventing or improving the cognitive dysfunction of older persons in the community, and further longitudinal study is warranted.

## Background

China has the largest number of older people in the world, and the proportion of older adults was also relatively high. As the number of older adults increases, the problem of the health of older adults is becoming the hotspot of the medicine field. Cognitive function refers to multiple mental abilities that include learning, thinking, reasoning, remembering, problem-solving, decision making, and attention [1]. In China, a large cohort study indicated that the prevalence of subjective cognitive decline for adults aged 60 to 80 years was 18.8% [2]. A national cross-sectional study showed that the prevalence of mild cognitive impairment for adults aged 60 years or older was 15.5% (approximately 38.8 million people) [3]. Cognitive decline has a negative impact on overall health, which imposes a heavy burden on the families and health service system [4, 5].

Regular exercise or physical activity is beneficial to promote physical and mental health, and reduce the occurrence and development of many chronic diseases. Recent studies indicated that physical activity can also increase cerebral blood flow, reduce brain shrinkage, improve brain tissue metabolism, promote the establishment of brain neural network and stimulate the central nervous system excitement, which may lead to adaptive changes in brain structure and function [6, 7]. In terms of the effect of physical exercise on cognitive function in older persons, a large number of randomized controlled studies have shown that aerobic exercise, resistance exercise, and aerobic combined resistance exercise have positive effects on cognitive function in older adults [8–10]. It was indicated that participating in physical exercise or physical activity may be an important protective factor for cognitive function in older persons. Besides, different types of exercise and the intensity of exercise also had an effect on the improvement degree of cognitive function [11].

It is known that physical fitness consists of several components including muscle strength, muscle endurance, agility, flexibility, balance, and cardiopulmonary endurance. Although the association of physical fitness with cognitive function has been proved [12–14], how many and how well the physical fitness components are linked to cognitive function in a given population is not clear. Therefore, the purpose of this study was to determine the association of physical fitness with cognitive function, and find out which aspects of physical fitness components are the most closely connected with cognitive function in community-dwelling older adults.

## Methods

### Participants

This research was conducted in Ningbo city, Zhejiang Province of China, from March to July 2019. Using a convenience sampling method, older participants in the study were recruited through community health service centers. The inclusion criteria for the study were that the participant (1) lived in communities, (2) aged 60 years old and over, (3) could communicate normally, and the mental conditions allowed them to cooperate to complete the cognitive function questionnaire test, (4) was able to complete the physical fitness measurements and (5) agreed to take part in the research. The exclusion criteria for the study were that the participant (1) had coronary heart disease, myocardial infarction, stroke or other cardiovascular diseases and (2) had musculoskeletal diseases such as lower limb arthritis, joint and muscle pain that affect human movement.

**Fig. 1:**
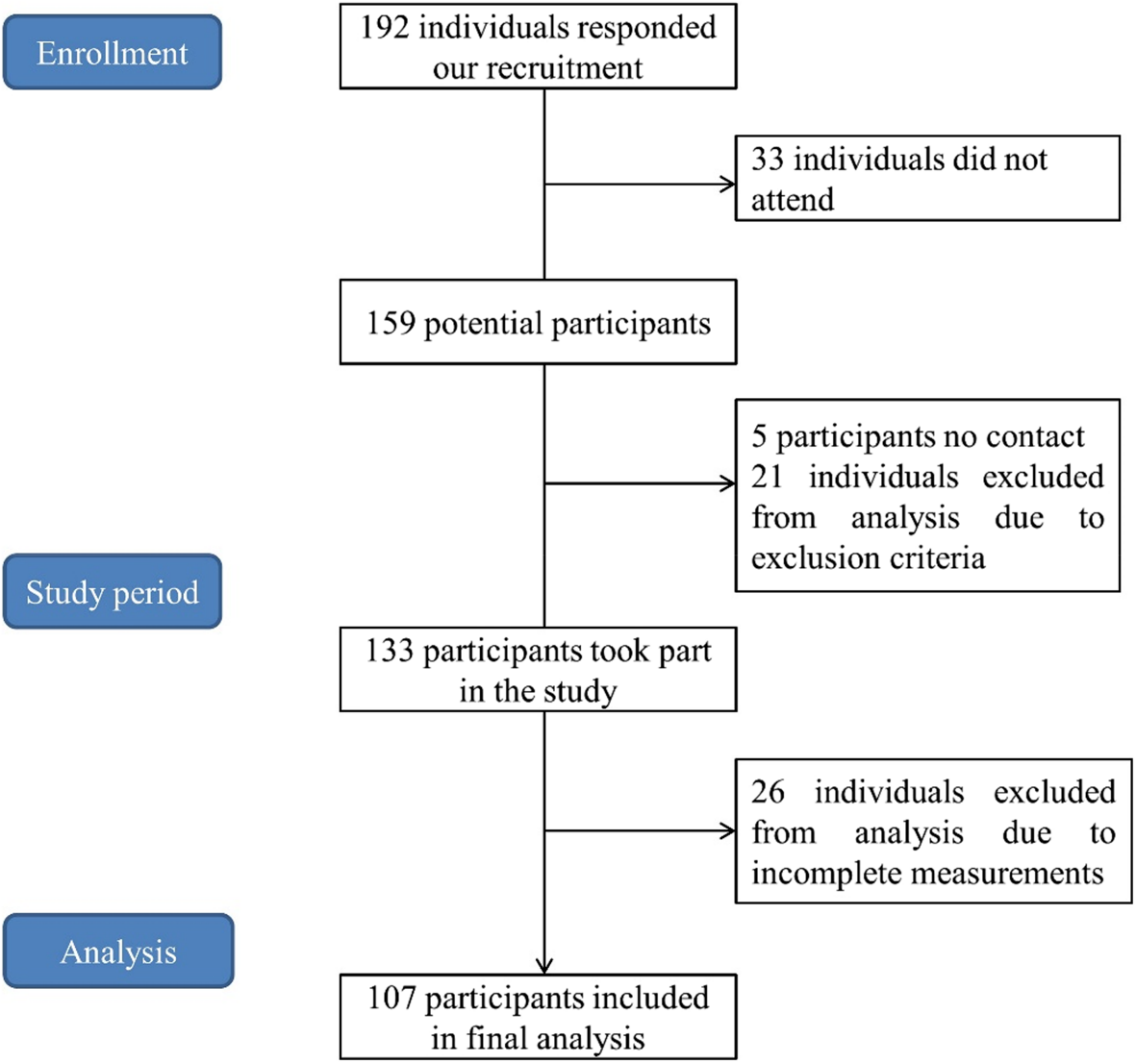
Flow diagram of participants inclusion

A total of 192 older people responded to the recruitment, 33 of them could not be accessed, 5 people could not be contacted, 21 of them were excluded due to exclusion criteria, and 26 people were excluded from analysis due to incomplete measurements, as a result of which the study was conducted with the participation of a total of 107 individuals (32 males and 75 females) (Fig. 1). Prior to the measures, the procedures and purposes of this research were well explained to each participant, and then the participant was asked to sign a written informed consent form. This study was reviewed and approved by the Human Ethics Board of Ningbo University.

### Cognitive function measurements

A Chinese version of the Mini-Mental State Examination (MMSE) was used to measure the cognitive function of older people in the community by a face-to-face questionnaire survey. The MMSE was used to measure subjects' ability of orientation, memory, registration, attention and calculation, recall, language and praxis. There are a total of 30 questions in the MMSE, and each question has a score. The score of the MMSE ranges from 0 to 30. According to the reference criteria of the MMSE in China [15], when the MMSE score is less than 27, the subject will be judged to have cognitive dysfunction or cognitive impairment. MMSE assessments were completed by several psychologists.

### Anthropometric measurements

Anthropometric indicators including body weight, height, and body mass index (BMI) were assessed by experienced graduate students. Each participant’s body weight was measured to the nearest 0.1 kg (kg), and body height was assessed to the nearest 0.1 cm (cm). Subsequently, BMI (kg/m^2^) was calculated as body weight (kg) divided by height in meters squared (m^2^).

### Physical fitness measurements

Physical fitness was assessed by measuring muscle strength, muscle endurance, agility, flexibility, balance, and cardiopulmonary endurance. Referring to previous studies [16–20], several test items were selected, including grip strength, 5-repetition sit-to-stand, timed up and go, sit and reach, one-leg balance with the eye open, and 6-min walk. In the study, the above test items were measured in a random order by experienced graduate students.

Grip strength: Each participant was asked to use the preferred hand to hold a dynamometer (Grip-D5101, Takei, Japan), and then asked to clench the dynamometer’s handle with maximum strength. Two trials were given for the test and there were 30 s (s) to rest between trials. The better reading to the nearest kilogram (kg) was used for further analysis.

5-repetition sit-to-stand: Participants were instructed to sit on a chair without armrests (45-cm height) until their feet were flat on the ground. They were asked to stand up until the hip and knee joints were extended fully and then sit down with their hands across the chest. The participants were instructed to perform this action 5 times as fast as possible. Two attempts were given, and the shorter time in seconds was taken for data analysis.

Timed up and go: The participant sat on a chair (45-cm height) with the arm resting on the lap or at the body sides comfortably (not on the armrests) and the hip positioning to the back of the seat all the way. The time it took for the participant to complete a set of movements included standing up from the chair, walking a 3-m distance, turning around, walking back, and sitting on the chair again. Two attempts were given for this test, and the shorter time in seconds was taken for data analysis.

Sit and reach: Participants were asked to take off shoes and sat on the ground with their legs stretched out and their feet flat on the front of the test instrument. Then, the participant was asked to slide their hand up the ruler as far as possible with their knees straight. There were 2 trials for this test, and the longer distance in centimeters was recorded for further analysis.

One-leg balance with the eye open: Each participant was requested to open the eyes and use the preferred foot stood and the other foot left the ground to keep balance with hands touching the waist. The number of seconds was recorded between the time the non-preferred foot left the ground and the time the balance lost. There were 2 attempts for the test, and the longer time in seconds was taken for data analysis.

6-min walk: Participants were requested to sit on chairs for about 5 min to rest at the departure area before the test. They were told to walk but not run for 6 min. In testing, the tester encouraged participants to walk as soon as possible and informed them of the consumed time in each minute. Only one attempt was given for the test. Walking distance in meters was measured and recorded for further analysis.

### Statistical analysis

Descriptive statistics was used to show an overview of the dataset. All the measured MMSE scores and physical fitness items are displayed as means with standard deviations (SD). The correlation between the cognitive function and the physical fitness of older adults was analyzed by partial correlation adjusting for sex, age, and years of education. Then, an analysis of covariance was used to compare the differences in physical fitness between older persons with or without cognitive impairment which is based on the MMSE score, adjusting for sex, age, and years of education. At last, multiple linear regression analysis was employed to obtain the main components that can predict the cognitive function of older people in the community. The MMSE score was set as the dependent variable, and physical fitness variables that had a level of significance less than 0.10 in the univariate analysis and that were considered to affect cognitive function including sex, age, and years of education, were added as independent variables. To obtain variables that were retained in the final model, the stepwise method was employed in the regression analysis. Data analysis was executed with IBM Statistical Package for Social Sciences (SPSS version 22.0; SPSS Inc., Chicago, IL, USA), with the level of statistical significance set at *P* < 0.05.

## Results

A total of 107 older people in the community were included in the final analysis. Descriptive characteristics of the participants were summarized in Table 1. The mean age was 71.7 ± 5 years, the mean BMI was 23.6 ± 2.6 kg/m^2^, and the mean years of education was 8.0 ± 3.4 years.

**Table 1:** Descriptive characteristics of the participants

	Male (*n* = 32)	Female (*n* = 75)	Total (*n* = 107)
Age (yrs)	72.8 ± 5.2	71.5 ± 5.5	71.7 ± 5.0
Height (cm)	161.6 ± 7.3	155.7 ± 6.0	158.3 ± 11.4
Weight (kg)	63.4 ± 13.4	57.2 ± 8.6	60.4 ± 8.2
BMI (kg/m^2^)	23.4 ± 2.1	24.7 ± 2.7	23.6 ± 2.6
Education (yrs)	8.8 ± 2.6	7.6 ± 4.7	8.0 ± 3.4

Association between physical fitness and MMSE scores in older adults was shown in Table 2. After adjusting for sex, age, and years of education, it was found that there was a positive relationship between grip strength, 6-min walk and MMSE scores (*r* = 0.42 and *r* = 0.35; *P* < 0.05), while there was a negative correlation between 5-repetition sit-to-stand and MMSE scores (*r* = -0.43, *P* < 0.01). However, no significant association was observed between timed up and go, sit and reach, one-leg balance with the eye open, and MMSE scores.

**Table 2:** Association of physical fitness with MMSE scores in older adults (adjusted sex, age and years of education)

	Grip strength	5-repetition sit-to-stand	Timed up and go	Sit and reach	One-leg balance with eye open	6-min walk
r	0.42	-0.43	-0.21	0.11	0.08	0.35
*P*	< 0.01	< 0.01	0.07	0.21	0.30	0.03

According to the scores of MMSE, the older participants in the community were divided into the group with cognitive impairment (MMSE score < 27, *n* = 25) and the group with normal cognitive function (MMSE score ≥ 27, *n *= 82) as shown in Table 3. The grip strength and 6-min walk in the cognitive impairment group was 24.5 ± 3.4 kg and 423.2 ± 53.7 m, which was significantly lower than that in the normal cognitive function group (26.8 ± 2.1 kg, 463.2 ± 33.9 m; *P* < 0.05). The 5-repetition sit-to-stand and timed up and go in the cognitive impairment group was significantly higher than that in the normal cognitive function group (8.4 ± 1.6 s vs. 7.3 ± 0.7 s, 6.8 ± 2.5 s vs. 6.0 ± 2.1 s; *P* < 0.05). However, no significant differences were found in sit and reach, one-leg balance with the eye open between the two groups.

**Table 3:** Physical fitness between older adults with different cognitive function level

	MMSE scores < 27 (*n* = 25)	MMSE scores ≥ 27 (*n* = 82)
Grip strength (kg)	24.5 ± 3.4	26.8 ± 2.1^*^
5-repetition sit-to-stand (s)	8.4 ± 1.6	7.3 ± 0.7^*^
Timed up and go (s)	6.8 ± 2.5	6.0 ± 2.1^*^
Sit and reach (cm)	8.0 ± 7.3	8.7 ± 9.2
One-leg balance with eye open (s)	4.8 ± 5.8	6.7 ± 4.3
6-min walk (m)	423.2 ± 53.7	463.2 ± 33.9^*^

^*^Represent significant differences between older adults with MMSE scores < 27 and MMSE scores ≥ 27

Multiple linear regression analysis was employed to obtain the main components that can explain the cognitive function of older adults. The dependent variable was set as MMSE scores, and the independent variable was set as items of physical fitness and other covariates such as sex, age, and years of education. As shown in Table 4, age, together with grip strength and 6-min walk can predict the cognitive function of older persons in the community (*P* < 0.05), which could explain 42% of the variation in the dependent variable (MMSE scores).

**Table 4:** Stepwise regression analysis between MMSE scores and measured variables

Variables	B	SE (B)	β	t	*P*	R^2^
(Constant)	-16.46	3.14		-6.20	< 0.01	0.42
Age	0.65	0.04	0.70	13.62	< 0.01	
Grip strength	0.87	0.12	0.36	7.31	< 0.01	
6-min walk	-0.06	0.03	-0.15	-2.13	0.02	

## Discussion

The cognitive function generally declines with the aging process. The purpose of this study was to explore the association of physical fitness with cognitive function, and find out which aspects of physical fitness components are the most closely related to cognitive function in older adults in the community. Results showed that the correlation analysis revealed that the grip strength and 6-min walk were positively related to cognitive function, while the 5-repetition sit-to-stand was negatively associated with cognitive function. The mean values of physical fitness including grip strength and 6-min walk were significantly lower, and 5-repetition sit-to-stand and timed up and go were significantly greater in older adults with cognitive impairment than those in the normal older adults. Stepwise regression analysis indicated that age, together with physical fitness items including grip strength and 6-min walk can explain the cognitive function in older adults.

Age-related loss of skeletal muscle mass and reduced muscle strength (sarcopenia) is associated with the occurrence of late-life cognitive impairment. A prospective cohort study with an average of 5.6-year follow-up showed that more severe sarcopenia at baseline was connected with a faster rate of cognitive decline, a higher risk of incident mild cognitive impairment and Alzheimer's dementia [21]. Sarcopenia has been reported to mediate the association between functional decline and cognitive impairment in cognitively impaired older persons [22]. Raji et al. [23] discovered that older adults with poor cognition have significantly lower handgrip strength compared to those with good cognition through a 7-year prospective cohort study. Similarly, in our study, handgrip strength was found to be positively associated with cognitive function.

Cardiopulmonary endurance is one of the most extensively studied items of physical fitness in older persons with poor cognition and is a predictor for cognitive decline [24]. Our study showed a positive relationship between cardiopulmonary endurance (6-min walk) and cognitive function. The result is similar to those reported by Sampaio and collaborators [14]. They carried out a cross-sectional study of 102 older people and found that cardiopulmonary endurance is the component with a greater relationship with cognitive function. Previous studies suggested that greater cardiopulmonary endurance is generally connected with neurovascular plasticity, neurogenesis and upregulation of neurotrophins that contribute to better brain health [7, 25].

Good physical fitness is the basis and premise for older adults to maintain their independence and keep healthy. Physical fitness generally includes muscle strength, muscle endurance, movement speed, agility, flexibility, balance, and cardiorespiratory endurance. A large number of studies have pointed out that physical fitness is closely related to the occurrence and development of many chronic diseases in older adults, such as hypertension, diabetes, non-alcoholic fatty liver disease, cardiovascular disease, and so on [26, 27]. Furthermore, results from an animal experiment showed that physical fitness is also closely related to brain structure and metabolism in rats [28]. The decline of physical fitness in older persons generally presents as the lower of muscle mass and muscle strength, the reduction of movement speed and agility, and the decrease of cardiopulmonary function, which may be linked with the decline of cognitive function.

On the other hand, study results from a German scholar who measured physical fitness and cognitive function of 72 older people with an average age of 69-year–old indicated that muscle strength, cardiopulmonary function, movement speed, flexibility, agility, and body balance were associated with cognitive function in older adults [29]. The previous results are both similar and different from the results of our study. The similarity lies in that muscular strength, muscular endurance, and cardiopulmonary endurance test indicators, such as grip strength, 5-repetition sit-to-stand, and 6-min walk were also observed to have significant correlation relationships with cognitive function in our study. While the difference lies in that our study had not found remarkable correlations between timed up and go, sit and reach, one-leg balance with the eye open, and cognitive function. The inconsistency with the previous research results may be caused by the varied body shape of subjects, and the different test indicators and test methods.

Aerobic exercise can promote cognitive function in older adults. A meta-analysis was conducted to investigate the effect of aerobic exercise on the cognitive function of elderly people in China [30]. This study found that aerobic exercise, such as rhythmic dancing, ergometer cycling, brisk walking, jogging, and square dancing could improve the cognitive function of elderly people. In addition, resistance exercise can also improve cognitive function in older adults. A study from a clinical trial showed that 12 weeks of momentum-based dumbbell training significantly improved overall cognitive function, executive function, memory, and attention in older patients [31]. Compared with aerobic exercise or resistance-resistance exercise alone, the combined model that includes two or more interventions such as physical exercise, dietary improvement, cognitive training, and social support may be better to prevent or treat cognitive impairment in older adults than the single model [32, 33].

Regular exercise or physical activity can improve cognitive function in older adults. The American Academy of Neurology encourages people with cognitive impairment to engage in physical activity that interests them, and it also points out that undertaking physical activity two or more times a week is beneficial to improve symptoms of cognitive impairment in older persons [34]. Moreover, previous studies showed that more than 90 min of moderate-to-vigorous intensity physical activities per week can promote the cognitive function of older patients [35–37]. Generally, an increase in physical activity inevitably leads to an increase in physical fitness. This may be one of the reasons why there was an association between physical fitness and cognitive function in the community-dwelling older adults in our study.

The potential mechanisms behind the association between physical activity or physical fitness and cognitive function are unclear. However, it is generally believed that physical activity can enhance the production of many kinds of growth factors such as insulin-like growth factor 1 and brain-derived neurotrophic factor, and lower inflammation [38–40]. These growth factors play an essential role in regulating the effects of physical activity on learning. This implies that low physical fitness level may be a major modifiable risk factor for poor cognitive function in older persons.

This study has some limitations in the following aspects. Firstly, there are many factors that affect the cognitive function of older adults. We only considered the influence of sex, age, and education level on cognitive function, and other potential confounders such as physical activity and diet were not investigated. Moreover, the results of this study were based on the data of older persons in the community. Therefore, whether the study results could also be applied to other populations, such as the seniors in care or nursing homes, remains to be verified. Thirdly, cognitive function was assessed only by MMSE. The cognitive assessment may be not sensitive to subtle cognitive impairment compared to other cognitive assessments such as the Montreal Cognitive Assessment. Fourthly, we acknowledge that false-positives (type I error) might occur when conducting multiple tests. The number of tests performed considering predictors could be due to the false-positives. Finally, this study analyzed the correlation between physical fitness and cognitive function, rather than the causal relationship. Therefore, longitudinal studies are needed to determine the causal relationship between physical fitness and cognitive function.

## Conclusions

The aim of the study was to investigate the association of physical fitness with cognitive function, and find out which aspects of physical fitness components are the most closely connected with cognitive function in older adults in the community. The main findings of this study suggest that there is an association between physical fitness and cognitive function, and the grip strength and 6-min walk can help explain the cognitive function in community-dwelling older adults. More attention needs to be paid to the increase in physical fitness for preventing or improving the cognitive dysfunction of older people in the community, and further longitudinal study is warranted.

## Data Availability

The datasets used and/or analyzed during the current study are available from the corresponding author upon reasonable request.

## Abbreviations

BMI: Body Mass Index
Cm: Centimeter
Kg: Kilogram
M: Meter
MMSE: Mini-Mental State Examination
S: Seconds
SD: Standard Deviation
